# An accelerometer-based dataset for monitoring slag in steel manufacturing

**DOI:** 10.1186/s13104-025-07486-8

**Published:** 2025-10-03

**Authors:** Mert Sehri, Lucas Mantuan Ayres, Francisco de Assis Boldt, Patrick Dumond, Marco Antonio de Souza Leite Cuadros

**Affiliations:** 1 Department of Mechanical Engineering, University of Ottawa, 161 Louis Pasteur, Ottawa, ON K1N 6N5 Canada; 2 Gaintech Tecnologia, Serra, ES 29166-845 Brazil; 3 Department of Informatics, Campus Serra do Instituto Federal do Espírito Santo (IFES), Serra, ES 29166-630 Brazil; 4 Department of Industrial Automation, Campus Serra do Instituto Federal do Espírito Santo (IFES), Serra, ES 29166-630 Brazil

**Keywords:** Slag flow, Steel manufacturing, Vibration, Condition monitoring, Signal processing

## Abstract

**Objectives:**

Slag detection in steel manufacturing is essential for ensuring high product quality and process efficiency. The purpose of the accelerometer-based data is to allow for accurate monitoring and differentiation between slag and molten metal flow. This is vital to prevent equipment damage, maintain steel quality, and enhance operational effectiveness. The data is collected specifically to support the development of machine learning models for real-time monitoring in the steel production process, addressing the critical need for precise slag detection.

**Data description:**

The Steel Slag Flow Dataset (SSFD) offers a comprehensive set of data obtained from a triaxial accelerometer during various stages of steel production. By leveraging this dataset, researchers can effectively analyze and classify the flow of slag versus molten metal. The dataset allows for data-driven approaches so that machine learning researchers can optimize steel manufacturing processes, ensuring high-quality steel production and minimizing the risks associated with slag contamination. The SSFD provides a valuable resource for researchers seeking to enhance predictive maintenance and monitoring in industrial applications.

## Objective

In a steel foundry, a vibration sensor is used to track flow changes in real time during production, collecting data to detect when slag movement begins and stops. This makes it possible to precisely follow the various stages of production and helps distinguish between molten metal and slag. The dataset created here encourages researchers to identify and categorize slag flow events in steel casting and forming processes using both traditional and deep learning techniques. This data makes it possible to use a variety of techniques and algorithms to determine the presence and patterns of slag flow during the production process. This dataset will contribute to improving machine learning model accuracy and encourages the development of various slag flow detection approaches by supporting the training and validation of deep learning algorithms [1–3]. The dataset’s potential in real-world slag monitoring applications is ensured by its incorporation of various conditions, such as fluctuating flow rates.

The Steel Slag Flow Dataset (SSFD) [4] was collected to address critical challenges in slag detection during steel manufacturing. This data serves the purpose of enabling accurate differentiation between slag and molten metal flow to prevent equipment damage, improve steel quality, and enhance the overall efficiency of the production process. Unlike traditional methods, this dataset supports the development of advanced machine learning models for real-time slag detection, which is crucial for modern industrial applications.

The dataset was not published as part of a standalone research paper but rather as a resource aimed at facilitating broader studies in vibration-based steel flow monitoring. This aligns with the growing need for open data that encourages innovation in slag flow analysis. No other publicly available slag flow dataset currently exists in the literature.

## Data description

An accelerometer with a sampling rate of 6,400 Hz was selected to capture the signal, where each file contains 5 s of data. The applicable frequency range is 3,200 Hz by applying the Shannon-Nyquist theorem [5, 6]. The data collection time is set to 5 s to have sufficient samples to distinguish between different flow conditions. Thus, 32,000 data samples are collected for each set of data. Each slag flow condition has varying vibrational amplitudes due to the use of different steel mixtures during slag pouring, enabling a variety of different cases. A time series sample of E-5 (early no slag), B-5 (before slag flow), and S-5 (during slag flow) is shown in Data File 1 (vibration data). The accelerometer sensor is placed at the operator end of the shaft that controls the molten steel flow, as shown in Data File 2, to reduce noise contained within the signal and protect the sensor from heat damage during production in industry.

Data is collected at a steel company. Data File 2 shows an image captured during the data collection process, where molten steel is getting poured into a cast. In the figure, the accelerometer sensor (PCB, model 356A15) is attached to the end of the flow control shaft (operator end) which extends past the right-hand side of the image. This setup allows vibration signatures to be collected for different slag flow conditions at the operator end of the system.

In each dataset file (in Table 1), the first column provides time data, whereas the second, third, and fourth columns are the x, y, and z axis of the accelerometer data, respectively. The raw data is provided as time series vibration amplitudes. The format of each set of data is as follows: {Letter}-{Number}. The first letter in the dataset labeling scheme is the slag flow condition. Specifically, the first letter can be denoted as “E” for early no slag (i.e. early case), “B” for before slag flow (i.e. just before the flow of slag), and “S” for during slag flow. The number in the dataset labeling scheme identifies different slag occurrences. For example, the data sample labeled “E-5” corresponds to a no slag passing for occurrence 5. Data File 3 in Table 2 provides a rundown of the dataset’s labels. Each occurrence contains a different slag mixture, causing a different slag flow rate. However, due to the company’s confidentiality requirements, material mixtures and flow specifications cannot be provided.

The accelerometer values are captured in $\:\text{V}$, and the data is then converted to $\:\frac{\text{m}}{{\text{s}}^{2}}$ using the sensitivity conversion rates provided by the manufacturer. Therefore, the data presented in each raw data file have vibration [accelerometer] units of $\:\frac{\text{m}}{{\text{s}}^{2}}$.

Table 1 provides a description and link to all the data files and datasets described herein.

**Table 1:** Overview of data files and datasets

Label	Name of data file/data set	File types (file extension)	Data repository and identifier (DOI or accession number)
Dataset	Steel Slag Flow Dataset (SSFD)	CSV files (.csv)	Mendeley Data (10.17632/mjnpsbs6fs) [4]
Data file 1	Figure 1 Accelerometer Data for E-5, B-5, and S-5	JPG file (.jpg)	Mendeley Data (10.17632/mjnpsbs6fs) [4]
Data file 2	Figure 2 Data Collection Process	JPG file (.jpg)	Mendeley Data (10.17632/mjnpsbs6fs) [4]
Data File 3	Table 2 Dataset Labelling for SSFD	JPG file (.jpg)	Mendeley Data (10.17632/mjnpsbs6fs) [4]

## Limitations

- The dataset provides a limited number of slag flow conditions. It is recognized that there can be many more. It provides 16 different slag mixture and flow conditions (the specifications of the conditions are confidential).
- Since no filter is applied, data preprocessing is likely necessary.

**Table 2:**
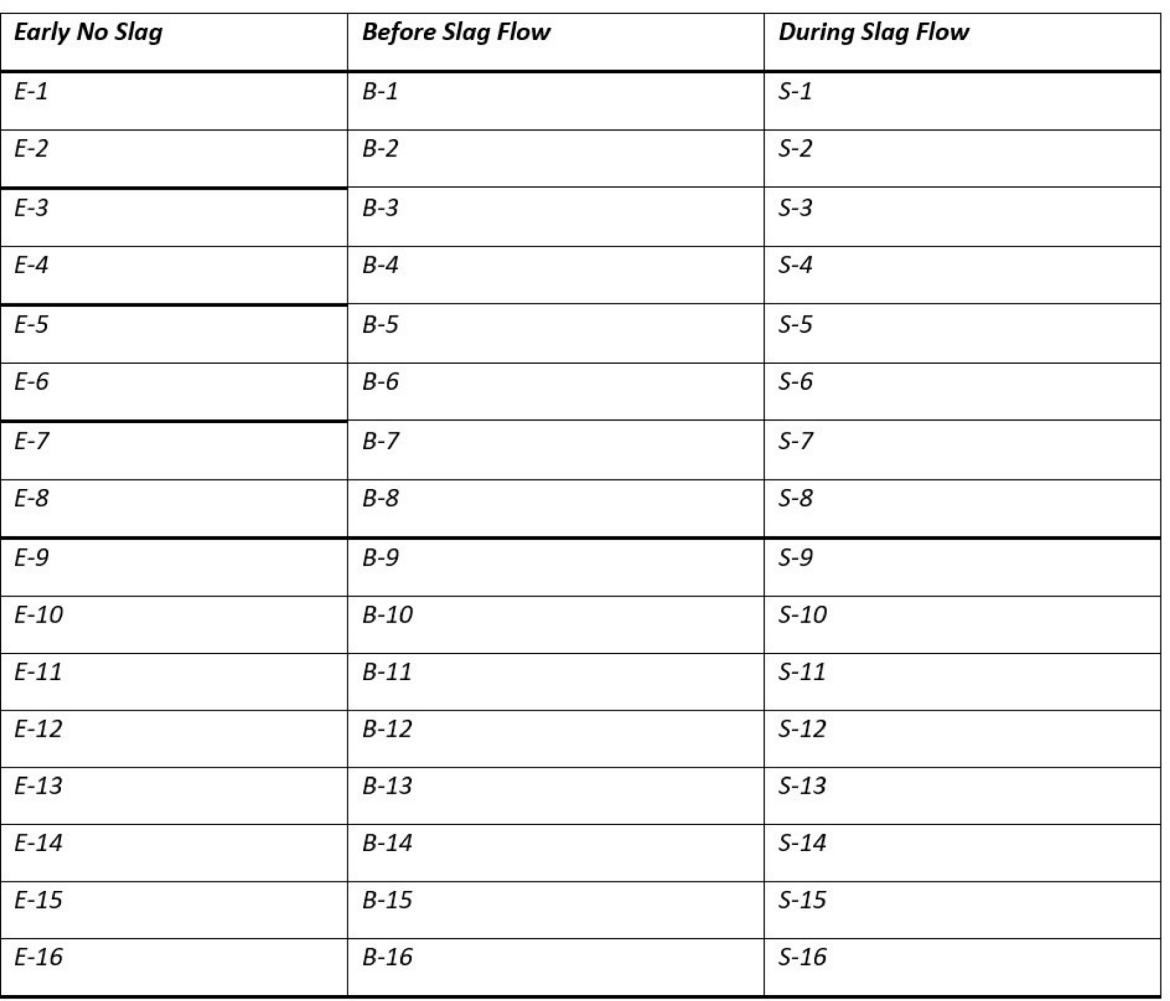
Dataset labelling for SSFD

**Fig. 1:**
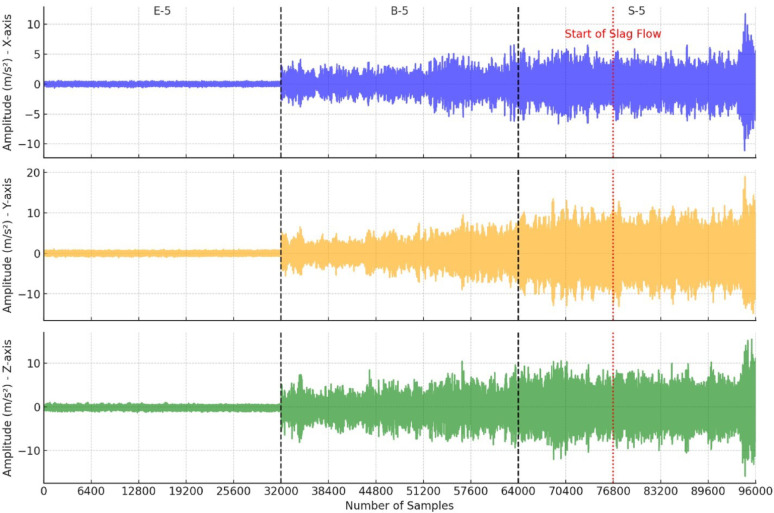
Accelerometer Data for E-5, B-6 and S-5

**Fig. 2:**
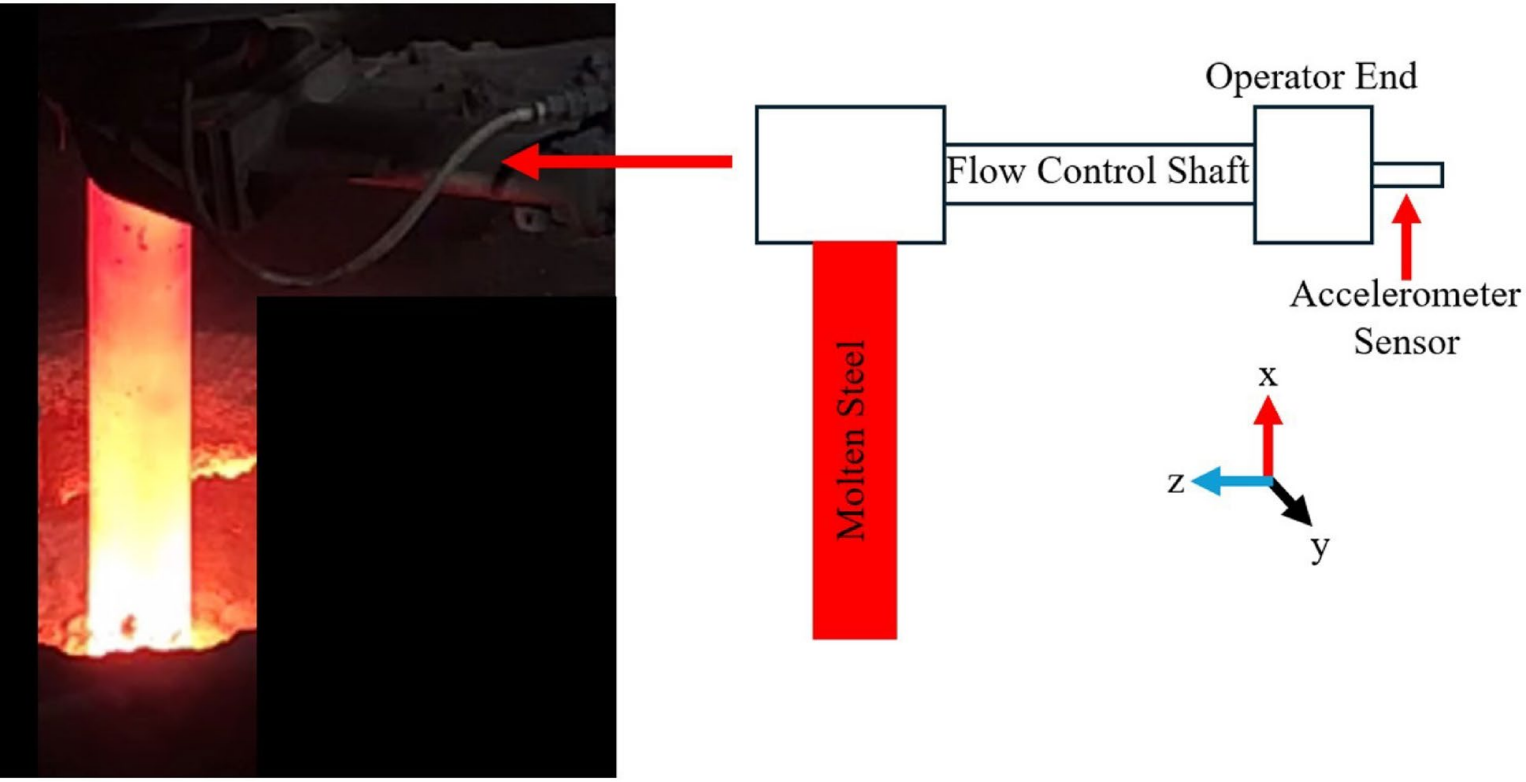
Data Collection Process

## Data Availability

Sequence data that support the findings of this study have been deposited in the Mendeley Data Repository at https://data.mendeley.com/datasets/mjnpsbs6fs.

## Abbreviations

SSFD: Steel Slag Flow Dataset
E-5: Early no slag for 5th flow condition
B-5: Before slag flow for 5th flow condition
S-5: During slag flow for 5th flow condition
